# Moderate and severe traumatic brain injury: lesion frequency distribution maps and prognostic importance of brain contusions on early MRI

**DOI:** 10.1007/s00330-025-12159-y

**Published:** 2025-12-19

**Authors:** Tiril Svaasand Eliassen, Lars Eirik Bø, Anne-Mari Holte Flusund, Anne Katharina Köster, Nada Andelic, Toril Skandsen, Anne Vik, Kent Gøran Moen

**Affiliations:** 1 Department of Circulation and Medical Imaging, Faculty of Medicine and Health Sciences, Norwegian University of Science and Technology (NTNU), Trondheim, Norway; 2 Department of Health Research, SINTEF Digital, Trondheim, Norway; 3 Department of Neuromedicine and Movement Science, Faculty of Medicine and Health Sciences, Norwegian University of Science and Technology (NTNU), Trondheim, Norway; 4 Department of Radiology, Møre and Romsdal Hospital Trust, Molde Hospital, Molde, Norway; 5 Institute of Health and Society, Research Centre for Habilitation and Rehabilitation Models and Services (CHARM), Faculty of Medicine, University of Oslo, Oslo, Norway; 6 Department of Physical Medicine and Rehabilitation, Oslo University Hospital, Ullevål, Norway; 7 Clinic of Rehabilitation, St. Olavs Hospital, Trondheim University Hospital, Trondheim, Norway; 8 Department of Neurosurgery, St. Olavs Hospital, Trondheim University Hospital, Trondheim, Norway; 9 Department of Radiology, Vestre Viken Hospital Trust, Drammen Hospital, Drammen, Norway; 10 Department of Radiology and Nuclear Medicine, St. Olavs Hospital, Trondheim University Hospital, Trondheim, Norway

**Keywords:** Neuroimaging, Brain contusion, Brain mapping, Patient outcome assessment

## Abstract

**Objective:**

Brain contusion frequency, distribution and prognostic value were investigated on early MRI in moderate-severe traumatic brain injury (TBI).

**Materials and methods:**

We prospectively included 301 patients (8–70 years) admitted to Trondheim or Oslo University Hospitals with moderate (*n* = 123) or severe (*n* = 178) TBI and brain contusion(s) on MRI within 6 weeks (median 9 days) post-injury. Volumetric segmentation of brain contusions was performed manually on fluid-attenuated inversion recovery (FLAIR) MRI. The segmentations were combined into lesion frequency distribution maps for the whole cohort, moderate and severe TBI separately and three outcome categories. The 12-month outcome was assessed with the Glasgow Outcome Scale-Extended (GOSE). Relationship with the outcome was evaluated visually, with adjusted analyses and with voxel-based lesion-symptom mapping (VLSM).

**Results:**

The frontal (75%) and temporal (82%) lobes had the highest frequency of brain contusions. There was no significant difference in total lesion volume between moderate and severe TBI (median: moderate TBI 15.8 cm^3^, severe TBI 13.6 cm^3^, *p* = 0.30). The total brain contusion volume significantly predicted the 12-month outcome in the adjusted models and decreased with increasing outcome category (GOSE score 1–4, median volume: 37.8 cm^3^; GOSE score 5–6, median volume: 14.5 cm^3^; GOSE score 7–8, median volume: 7.1 cm^3^; *p* = 0.02). There was no significant association between lesion location and the GOSE score in the VLSM analyses.

**Conclusion:**

In this MRI cohort study, we found similar distributions and volumes of brain contusions in moderate-severe TBI. The brain contusion volume significantly predicted the 12-month outcome, whereas the lesion location was not associated with the outcome.

**Key Points:**

***Question*** *Accurate prognostic models for TBI are important for clinical decision making, but the value of including MRI measures remains unclear.*

***Findings*** *In moderate and severe TBI, brain contusions had the highest frequency in frontotemporal regions. Contusion volume predicted 12-month functional outcome, whereas there was no association with location.*

***Clinical relevance*** *Our findings indicate that brain contusion volume is more important for functional outcomes than lesion location. The brain contusion volume should be included in future AI models as one predictor of patient outcome after TBI.*

**Graphical Abstract:**

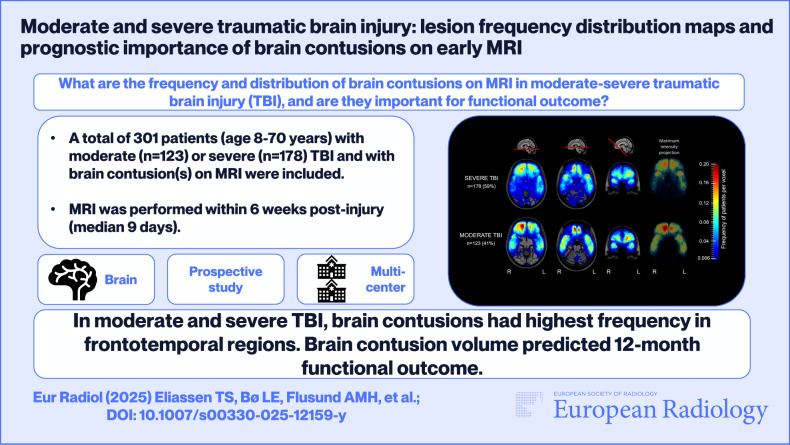

## Introduction

Brain contusion is a common lesion type in moderate and severe traumatic brain injury (TBI) [1] that occurs when the brain parenchyma strikes the skull or the dural folds. Frontal and temporal lobes are known to be predilection sites for brain contusions [1–3]. Current literature has shown that MRI findings have prognostic value in TBI [4], and when CT findings do not explain the clinical presentation of a patient, MRI is recommended, especially to clarify the extent of traumatic axonal injury (TAI) [5, 6]. Additionally, when it comes to brain contusions, MRI gives a more accurate visualization than CT because of its greater ability to detect non-hemorrhagic lesions [7–10].

Establishing good prognostic models for TBI is important for clinical decision-making and for providing patients and their families with reliable outcome predictions. The Corticosteroid Randomization after Significant Head Injury (CRASH) and International Mission for Prognosis and Analysis of Clinical Trials in Traumatic Brain Injury (IMPACT) models are the most validated prognostic models for TBI [11]. However, they have been shown not to accurately predict individual outcomes [12], and the additional predictive value of including MRI measures remains unclear. In a part of the patient cohort comprising this study, our research group has previously shown that the volume of brain contusions on MRI can significantly predict 12-month functional outcomes in patients with moderate TBI but not in patients with severe TBI [13]. However, it is not known whether the location of brain contusions is associated with outcomes.

In lesion mapping, segmented lesions from individual patients’ MRI scans are transferred to a common lesion frequency distribution map. In contrast to conventional statistics, lesion mapping enables us to visually study the distribution and frequency of brain contusions in a three-dimensional (3D) interactive viewer. In addition, statistical lesion maps, such as those resulting from voxel-based lesion-symptom mapping (VLSM), provide a quantitative method for analyzing associations between precise lesion distributions and clinical outcomes [14]. In MRI studies of brain contusions, lesion mapping has been performed in only two small studies (*n* < 35) without VLSM analyses [15, 16]. However, VLSM has been proven useful in studies of other pathologies such as strokes [14], high-grade gliomas [17] and TAI [18]. Hence, lesion mapping with VLSM can extend our knowledge of brain contusions and their importance for patient outcomes.

In this study, we used conventional statistics and lesion frequency distribution maps to analyze manually segmented brain contusions from individual early MRI scans of patients with moderate and severe TBI. The aims were (1) to evaluate the overall distribution and frequency of brain contusions with conventional statistics and on lesion frequency distribution maps in moderate-severe TBI and in moderate and severe TBI separately and (2) to investigate the prognostic value of brain contusions on fluid-attenuated inversion recovery (FLAIR) using both conventional statistics with regression models, lesion frequency distribution maps of outcome categories and VLSM.

## Materials and methods

### Ethics

The study was approved by the Regional Committee for Medical Research Ethics (2009/2328, 2017/1214). Consent was obtained from surviving patients eligible for follow-up, or for incapacitated or younger than 16 years, from their next of kin.

### Study population

Patients from two Norwegian prospective TBI cohorts (2014–2021) were included in the study: (1) the Trondheim moderate and severe TBI study (*n* = 673) [19], hereafter referred to as the Trondheim cohort, and (2) a severe TBI cohort from Oslo University Hospital (*n* = 153), hereafter referred to as the Oslo cohort [20]. The severity of the TBI was classified as moderate in patients with Glasgow Coma Scale (GCS) scores of 9–13 and as severe in patients with GCS scores ≤ 8 at admission or before sedation (Trondheim cohort) or at some time point during the first 24 h (Oslo cohort). For five patients, exact GCS scores were missing, and information from hospital records was used to classify TBI into moderate or severe.

**Fig. 1:**
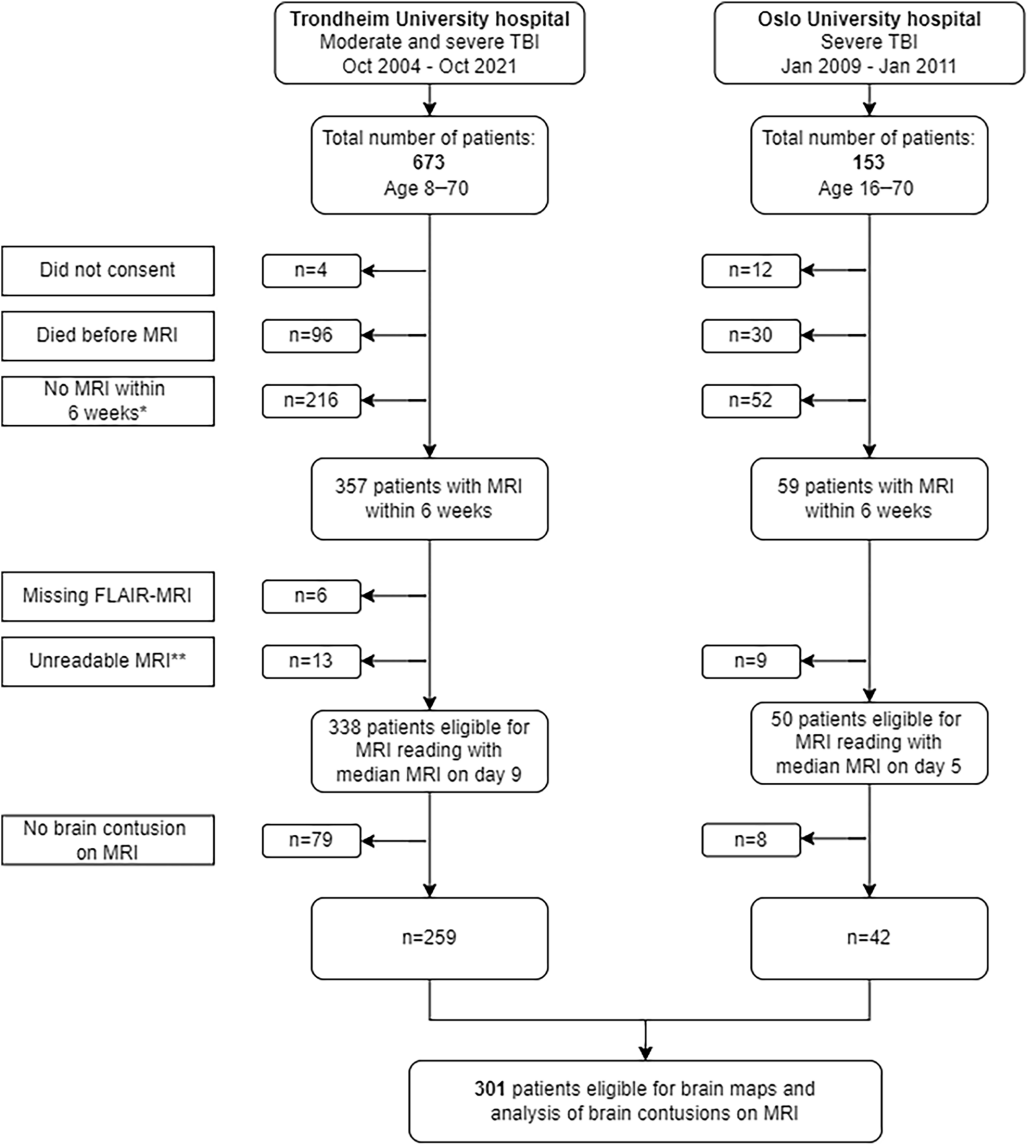
Flowchart of the two TBI cohorts and the 301 included patients. TBI, traumatic brain injury; MRI, magnetic resonance imaging; FLAIR, fluid attenuated inversion recovery * Due to attenutation of some traumatic lesions over time [21] ** Unreadable MRI due to poor quality, large artefacts, or missing sequences

A total of 301 patients with at least one brain contusion on FLAIR obtained within 6 weeks after injury were included in the study (Fig. 1). The exclusion criteria were (1) age > 70 years; or (2) age < 8 years; or (3) MRI obtained more than 6 weeks after injury due to attenuation of some traumatic lesions over time [21]; or (4) unreadable MRI (see Supplementary Material for more details).

### Neuroimaging, MRI reading and segmentation of brain contusions

All patients underwent CT as the first head examination. In this study, the worst scores of the CT scans were used because brain contusions often progress during the first 12 h post-injury [8, 22, 23].

MRI was obtained at field strengths 1.5 T (*n* = 258), 3 T (*n* = 36) or 1 T (*n* = 7). The MRI protocols included FLAIR, diffusion-weighted imaging (DWI), and T2*-weighted gradient-recalled echo (T2*GRE) or susceptibility-weighted imaging (SWI). For more information on the protocols, we refer to a previous study [24].

The MRIs were reviewed according to a predefined template developed as part of an international study on traumatic axonal injury (TAI), the TAI-MRI ERANET-NEURON [24]. Since the T2*GRE/SWI sequence overestimates hemorrhagic and underestimates non-hemorrhagic components and the DWI sequence has reduced tissue contrast, only segmentations from the FLAIR sequence were used for volume measurements of brain contusions.

Hemorrhagic and non-hemorrhagic components of the brain contusions were segmented separately with different labels so that they could be analyzed both separately and as complete lesions (hereafter referred to as total brain contusions). The brain contusions and TAI lesions were delineated manually using the open-source software 3D Slicer [25, 26] (version 4.8.0), and their presence, location, number, and volume were registered in predefined locations. The distribution of TAI lesions was registered with the standard TAI grade (grades 1–3) [27, 28] and the Trondheim TAI-MRI grade (grades 1–5) [13].

### Assessment of outcomes

The functional outcome was assessed by telephone or in person, using the structured interview for the Glasgow Outcome Scale Extended (GOSE) [29]. The GOSE score at 12 months was used because it is considered the best time point [30]. A total of 29 patients in the Trondheim cohort had missing 12-month GOSE scores. For seven of the patients, the 6-month GOSE score was used instead. 22 patients (7%) had missing GOSE scores at both 6 months and 12 months post-injury. For these patients, a model-based imputation algorithm was used [31, 32].

GOSE scores were divided into three categories: (1) severe disability, defined as a GOSE score of 1–4; (2) moderate disability, defined as a GOSE score of 5–6; and (3) good recovery, defined as a GOSE score of 7–8.

### Registration of segmented brain contusions and lesion frequency distribution map analyses

The FLAIR images with segmented brain contusions were spatially aligned with the standardized coordinate frame known as the Montreal Neurological Institute (MNI) space. This is defined by the ICBM-152 brain template, which is an average image based on 152 young normal brains [33]. First, the individual images were preprocessed and non-brain structures were removed using a deep learning model for automatic brain segmentation, which is publicly available through the open-source software Raidionics (https://github.com/raidionics/Raidionics-models/releases) [34]. The images were spatially aligned with the average brain by intensity-based, nonlinear image registration using the Advanced Neuroimaging Tools (ANTs) registration toolkit [35]. The segmented brain contusions were used to mask out the brain contusion region from the estimation of the nonlinear transformation. To bring all the brain contusions into a common space, the transformations were then applied to the individual brain contusion segmentations.

When all individual segmentations were registered to the MNI space, lesion frequency distribution maps displaying the segmented brain contusions were created for: (1) all TBI patients, (2) moderate and severe TBI patients separately, and (3) TBI patients in different outcome categories (GOSE scores of 1–4, GOSE scores of 5–6 and GOSE scores of 7–8). The frequency and distribution of brain contusions were studied visually on the lesion frequency distribution maps in 3D Slicer, version 4.8.0.

### Statistics

Continuous data were examined with the Mann–Whitney U test, and categorical data were analyzed using cross-tables and the Chi-square test or Fisher’s exact test. The Jonckheere-Terpstra test was used to analyze trends in the number and volume of brain contusions, the presence of TAI and the TAI grade between the outcome categories. To explore the effects of MRI field strength and the number of days from injury to MRI on brain contusion volume, we used the Spearman correlation test, ANOVA models and linear regression models. For more information on these analyses, we refer to the Supplementary Statistics section.

To explore the prognostic value of total and hemorrhagic brain contusion volume, we performed ordinal logistic regression models with outcome category (GOSE score 1–4, GOSE score 5–6 and GOSE score 7–8) as the dependent variable. Age, pupil abnormality, GCS score, number of days from injury to MRI and total (models 1 and 2) or hemorrhagic (models 3 and 4) brain contusion volume were used as covariates in all the models. In addition, models 1 and 3 included the TAI volume on FLAIR, whereas models 2 and 4 included the Trondheim TAI-MRI grade. Due to missing values, 10 patients (3%) were excluded from the models (GCS score, *n* = 5; pupil abnormality, *n* = 5). All the statistical analyses were performed with the software package IBM SPSS Statistics 28.0.1.0. The threshold for significance was set at *p* < 0.05.

VLSM was used to analyze the association between anatomical lesion distribution and functional outcome by using the NiiStat toolbox for Matlab (http://www.nitrc.org/projects/niistat). To increase the power, we excluded voxels with fewer than three overlapping lesions. For the remaining voxels, the GOSE scores of the patients with brain contusion in the given voxel were compared with the scores of the patients without lesions in the given voxel using the Student’s pooled-variance *t*-test with a *p* < 0.05 significance threshold. Finally, permutation thresholding with 2000 permutations was applied to correct for multiple comparisons.

More details on the materials and methods can be found in the Supplementary Methods.

## Results

### Patient and injury characteristics

Brain contusions were detected in 301 (76%) of the patients with early MRI (Fig. 1), of which only 68% had brain contusions on CT (Table 1). The included patients were older, more often suffered falls, had more CT findings and worse unadjusted outcomes than the excluded patients without brain contusions on MRI (*n* = 87) (Table 1).

**Table 1:** Patient and injury characteristics in the included and excluded MRI cohorts

	Patients with brain contusion(s) on MRI			Patients without brain contusion on MRI Moderate and severe TBI	*p*-value***
	Severe TBI*	Moderate TBI**	Moderate and severe TBI		
No.	178	123	301	87	
Age	28.5 (20.7–44.2)	32.5 (22.1–54.8)	32.0 (21.0–49.3)	24.7 (19.9–43.4)	**0.03**
Sex (male/female)	146/32 (82%/18%)	95/28 (77%/23%)	241/60 (80%/20%)	67/20 (77%/23%)	0.56
Injury mechanism					
Road traffic accident	97 (55%)	43 (35%)	140 (47%)	59 (68%)	**< 0.001**
Fall	62 (35%)	56 (46%)	118 (39%)	22 (25%)	**0.02**
Struck object/Violence	10 (6%)	8 (7%)	18 (6%)	2 (2%)	0.27
Other/Unknown	9 (5%)	16 (13%)	25 (8%)	4 (5%)	0.25
GCS score	6 (3–7)	12 (11–13)	8 (5–11)	9 (6–12)	0.17
Missing	3 (1.7%)	2 (1.7%)	5 (1.7%)	9 (10%)	
Pupil size at admission					
Normal size	120 (67%)	116 (94%)	236 (78%)	82 (94%)	**< 0.001**
Unilateral dilatation	45 (25%)	3 (2%)	48 (16%)	3 (3%)	**0.002**
Bilateral dilatation	11 (6%)	1 (1%)	12 (4%)	2 (2%)	0.54
Untestable or missing	2 (1%)	3 (2%)	5 (2%)	0 (0%)	0.36
Worst Marshall CT score	3 (2–5)	2 (2–5)	3 (2–5)	2 (1–2)	**< 0.001**
1	5 (3%)	8 (7%)	13 (4%)	24 (28%)	**< 0.001**
2	68 (38%)	68 (55%)	136 (45%)	49 (56%)	0.07
3 or 4	40 (23%)	12 (10%)	52 (17%)	11 (13%)	0.30
5 or 6	65 (37%)	35 (28%)	100 (33%)	3 (3%)	**< 0.001**
Worst Rotterdam CT score	3 (2–4)	3 (2–3)	3 (2–4)	2 (2–3)	**< 0.001**
1	4 (2%)	7 (6%)	11 (4%)	1 (1%)	0.31
2	41 (23%)	48 (39%)	89 (30%)	45 (52%)	**< 0.001**
3	58 (33%)	54 (44%)	112 (37%)	32 (37%)	0.94
4	47 (26%)	8 (7%)	55 (18%)	8 (9%)	**0.043**
5	21 (12%)	5 (4%)	26 (9%)	0 (0%)	**0.005**
6	7 (4%)	1 (1%)	8 (3%)	1 (1%)	0.41
Brain contusion(s) on CT (Yes)	125 (70%)	80 (65%)	205 (68%)	0 (0%)	**< 0.001**
Evacuated mass lesion§ (Yes)	51 (29%)	18 (15%)	69 (23%)	6 (7%)	**< 0.001**
Days from injury to MRI	10 (5–18)	6 (3–14)	9 (4–17)	8 (3–21)	0.35
Presence of TAI (Yes)	154 (87%)	84 (68%)	238 (79%)	74 (84%)	0.22ᵻᵻ
Standard TAI gradeᵻ	2 (1–3)	1 (1–2)	2 (1–3)	2 (1–3)	0.14ᵻᵻ
Trondheim TAI-MRI gradeᵻ	3 (2–4)	2 (1–3)	2 (1–3)	3 (2–3)	0.49ᵻᵻ
GOSE score#	5 (3–7)	7 (6–8)	6 (5–8)	7 (6–8)	**< 0.001**ᵻᵻ
1–4	54 (30%)	6 (5%)	60 (20%)	5 (7%)	**0.002**ᵻᵻ
5–6	67 (38%)	40 (33%)	107 (36%)	28 (32%)	0.56
7–8	43 (24%)	69 (56%)	112 (37%)	49 (56%)	**0.001**ᵻᵻ
Missing	14 (8%)	8 (7%)	22 (7%)	5 (6%)	

Values are presented as number of patients (%) or median (interquartile range (IQR: 25^th^–75^th^ percentile)) unless otherwise is indicated. *p*-values under 0.05 are indicated in bold

*MRI* magnetic resonance imaging, *TBI* traumatic brain injury, *No.* number, *GCS* Glasgow coma scale, *CT* computer tomography, *TAI* traumatic axonal injury, *GOSE* Glasgow outcome scale extended, *HISS* head injury severity scale

* Severe TBI: GCS ≤ 8. For 3 patients exact GCS score is missing but we were able to classify HISS category

** Moderate TBI: GCS 9–13. For 2 patients exact GCS score is missing but we were able to classify HISS category

*** Comparing moderate and severe TBI patients with brain contusions to moderate and severe TBI patients without brain contusions on MRI

§ Evacuated brain contusion/intracranial hematoma or extra axial hematoma at any time point during the hospital stay

# GOSE score was obtained 12 months after injury. 7 of the GOSE scores are from 6 months due to missing GOSE score at 12 months

ᵻ Standard TAI grade ranges from 1 to 3, while Trondheim TAI-MRI grade ranges from 1 to 5

ᵻᵻ *p* < 0.001 when comparing moderate and severe TBI

The patients with moderate TBI (*n* = 178) underwent MRI earlier (Table 2), had lower TAI grades and had better outcomes than those with severe TBI (*n* = 123) (Table 1).

**Table 2:** Lesion measures and distribution of brain contusions on early MRI in different injury severity groups based on individual segmentations

	Severe TBI*	Moderate TBI**	Moderate and severe TBI	*p*-value***
No.	178	123	301	
Days from injury to MRI	10 (5–18)	6 (3–14)	9 (4–17)	**0.01**
Total number of brain contusions on FLAIR-MRI	4 (2–7)	3 (2–6)	3 (2–6)	0.82
Total lesion volume of brain contusion on FLAIR-MRI (cm^3^)	13.56 (2.55–38.97)	15.79 (3.70–40.23)	13.76 (2.83–39.45)	0.30
Total volume of hemorrhagic component of brain contusion in FLAIR–MRI (cm^3^)	0.14 (0.00–3.02)	1.92 (0.00–7.52)	0.46 (0.00–4.10)	**< 0.001**
Probable affected eloquent cortical areas (yes)	60 (34%)	32 (26%)	92 (31%)	0.15
Motor cortex	21 (12%)	9 (7%)	30 (10%)	0.20
Sensory cortex	19 (11%)	10 (8%)	29 (10%)	0.46
Visual cortex	11 (6%)	3 (2%)	14 (5%)	0.13
Speech cortex	34 (19%)	17 (14%)	51 (17%)	0.23
Primary auditive cortex	1 (1%)	0 (0%)	1 (0%)	1
Number of patients with brain contusion(s) on FLAIR-MRI in:				
Frontal lobes	130 (73%)	96 (78%)	226 (75%)	0.32
Temporal lobes	149 (84%)	97 (79%)	246 (82%)	0.29
Parietal lobes	41 (23%)	25 (20%)	66 (22%)	0.58
Occipital lobes	17 (10%)	6 (5%)	23 (8%)	0.13
Cerebellum	13 (7%)	11 (9%)	24 (8%)	0.61
Deep structures#	12 (7%)	4 (3%)	16 (5%)	0.19
Brain stem	6 (3%)	2 (2%)	8 (3%)	0.48
Use of antithrombotic medications (Yes)	5 (3%)	4 (2%)	9 (3%)	0.86
Unknown	1 (1%)	1 (1%)	2 (1%)	
Evacuated mass lesion§ (Yes)	51 (29%)	18 (15%)	69 (23%)	**0.004**

Values are presented as number of patients (%) or median (IQR) unless otherwise is indicated. *p*-values under 0.05 are indicated in bold

*MRI* magnetic resonance imaging, *TBI* traumatic brain injury, *No.* number, *FLAIR* fluid attenuated inversion recovery, *GCS* Glasgow coma scale, *HISS* head injury severity scale

* Severe TBI: GCS ≤ 8. For 3 patients exact GCS score is missing but we were able to classify HISS category

** Moderate TBI: GCS 9–13. For 2 patients exact GCS score is missing but we were able to classify HISS category

*** Comparing moderate TBI patients to severe TBI patients

# Including corpus callosum, internal capsule, thalamus and basal ganglia

§ Evacuated brain contusion/intracranial hematoma or extra axial hematoma at any time point during the hospital stay. 7 patients had evacuated brain contusion/intracranial hematoma, of which 3 had a moderate TBI and 4 had a severe TBI

### MRI characteristics and brain contusion volumes

Patients imaged with 1.5-T and 3-T scanners had significantly larger brain contusion volumes than those imaged with 1-T scanners (Supplementary Tables 2, 3). According to the adjusted analyses, the number of days from injury to MRI was not associated with total brain contusion volume (Supplementary Tables 4, 5).

### Frequency and distribution of brain contusions in moderate and severe TBI combined

**Fig. 2:**
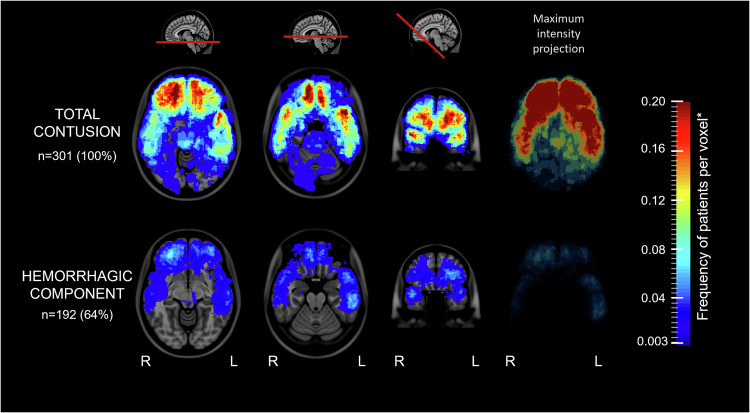
Lesion frequency distribution maps of total brain contusion versus the hemorrhagic component on FLAIR. Lesion frequency distribution maps showing the relative frequency of cases with total brain contusion (top) and a hemorrhagic component (bottom) at two transversal levels (level with the mesencephalon and pons), one oblique coronal plane and one maximum intensity projection. The color scale ranges from blue to red, where dark blue indicates at least one patient per voxel (~1%), and the darkest shade of red indicates a high percentage (up to 20%) of patients per voxel. For a more detailed presentation of the lesion frequency distribution maps, see the attached video “Video 1”. FLAIR, fluid-attenuated inversion recovery

**Fig. 3:**
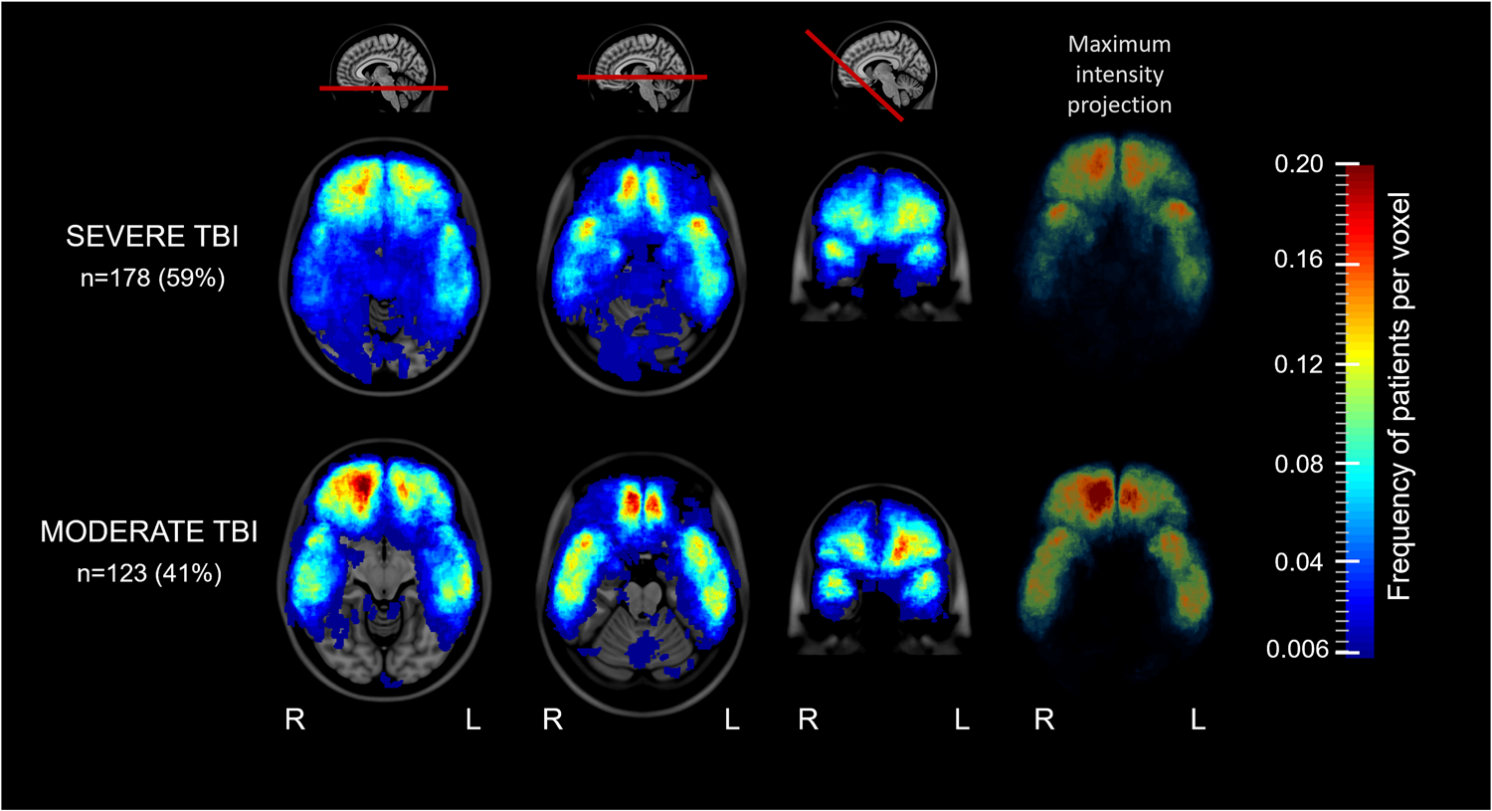
Lesion frequency distribution maps of brain contusions on FLAIR in severe versus moderate TBI. Lesion frequency distribution maps showing the relative frequency of brain contusions in different injury severity groups at two transversal levels (level with the mesencephalon and pons), one oblique coronal plane and one maximum intensity projection. The color scale ranges from blue to red, where dark blue indicates at least one patient per voxel (~1%) and the darkest shade of red indicates a high percentage (~20%) of patients per voxel. For a more detailed presentation of the lesion frequency distribution maps, see the attached video “Video 2”. FLAIR, fluid-attenuated inversion recovery; TBI, traumatic brain injury

Visual evaluation of all lesion frequency distribution maps revealed the highest frequency of brain contusions in the inferior and rostral parts of the frontal and temporal lobes (Figs. 2, 3, Videos 1, 2). In the frontal lobes, there was an increased frequency of brain contusions in the parasagittal area along the falx cerebri.

Statistical analysis of the individual segmentations revealed significantly more brain contusions in the left frontal lobe and in the right parietal and occipital lobes than in the opposite sides (Supplementary Table 1).

### Frequency and distribution of brain contusions in moderate versus severe TBI

Visual analyses of the lesion frequency distribution maps indicated a higher frequency of brain contusions in moderate TBI compared to severe TBI (Fig. 3, Video 2). However, the unadjusted statistical analyses of the individual segmentations did not reveal any significant differences in the distribution, total volume or number of brain contusions between the two injury severity groups (Table 2). However, patients with moderate TBI had significantly larger hemorrhagic brain contusion volumes than patients with severe TBI.

### Frequency and distribution of brain contusions in different outcome categories

**Fig. 4:**
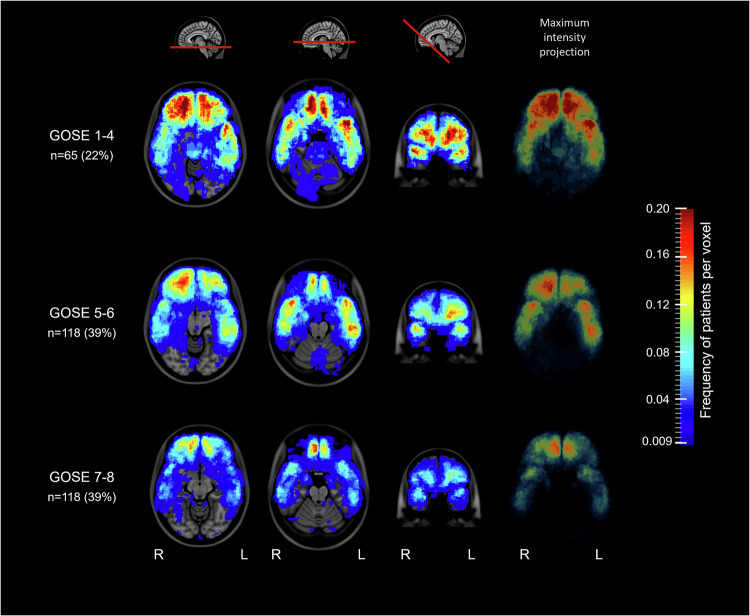
Lesion frequency distribution maps of brain contusions on FLAIR in different 12-month outcome categories. Lesion frequency distribution maps showing the relative frequency of brain contusions in different 12-month outcome categories (GOSE 1–4, *n* = 65; GOSE 5–6, *n* = 118; GOSE 7–8, *n* = 118) at two transversal levels (level with the mesencephalon and pons), one oblique coronal plane and one maximum intensity projection. The color scale ranges from blue to red, where dark blue indicates at least one patient per voxel (~1%) and the darkest shade of red indicates a high percentage (~20%) of patients per voxel. For a more detailed presentation of the lesion frequency distribution maps, see the attached video “Video 3”. FLAIR, fluid-attenuated inversion recovery; GOSE, Glasgow outcome scale extended

The lesion frequency distribution maps revealed a higher frequency of brain contusions in patients with severe disability at 12 months (GOSE scores of 1–4: *n* = 65, of which 91% had severe TBI) than in patients with moderate disability (GOSE scores of 5–6: *n* = 119) or good recovery (GOSE scores of 7–8: *n* = 117) (Fig. 4, Video 3). This finding was also supported by unadjusted analyses of the individual segmentations, which revealed a significant trend toward an increase in the number and total volume of brain contusions with a worse outcome category (Table 3).

**Table 3:** Lesion measures and distribution of brain contusions on early MRI in different outcome categories based on individual segmentations in moderate and severe TBI combined

	GOSE 1–4*	GOSE 5–6*	GOSE 7–8*	*p*-value**
No.	65	118	118	
Age	41.3 (24.5–59.5)	34.5 (22.5–50.5)	25.0 (18.6–40.9)	**< 0.001**
Injury severity (severe TBI ᵻ)	59 (91%)	76 (64%)	43 (36%)	
Days from injury to MRI	12 (7–20)	9 (5–17)	6 (3–13)	**0.006**
Total number of brain contusions on FLAIR-MRI	5 (3–8)	3 (2–7)	3 (2–5)	**< 0.001**
Total lesion volume of brain contusion on FLAIR-MRI (cm^3^) (median, IQR)	37.75 (12.93–86.27)	14.48 (2.89–42.79)	7.09 (2.27–21.80)	**< 0.001**
Total volume of hemorrhagic component of brain contusion in FLAIR-MRI (cm^3^) (median, IQR)	1.42 (0.00–6.43)	0.49 (0.00–4.33)	0.29 (0.00–2.40)	0.06
Number of patients with brain contusion(s) on FLAIR-MRI in:				
Frontal lobes	56 (86%)	88 (75%)	82 (70%)	**0.02**
Temporal lobes	55 (85%)	102 (86%)	89 (75%)	0.07
Parietal lobes	27 (42%)	24 (20%)	15 (13%)	**< 0.001**
Occipital lobes	11 (17%)	7 (6%)	5 (4%)	**0.004**
Cerebellum	5 (8%)	9 (8%)	10 (9%)	0.83
Deep brain structures#	10 (15%)	4 (3%)	2 (2%)	**< 0.001**
Brain stem	5 (8%)	2 (2%)	1 (1%)	**0.01**
Presence of TAI (yes)	56 (86%)	93 (79%)	89 (75%)	0.11
Standard TAI grade (mean, SD)	2.53 (0.69)	1.95 (0.84)	1.62 (0.77)	**< 0.001**
Trondheim TAI-MRI grade (mean, SD)	3.29 (1.19)	2.39 (1.22)	1.98 (0.98)	**< 0.001**

Values are presented as number of patients (%) or median (IQR) unless otherwise is indicated. *p*-values under 0.05 are indicated in bold

*GOSE* Glasgow outcome scale extended, *No.* number, *FLAIR* fluid attenuated inversion recovery, *MRI* magnetic resonance imaging, *TAI* traumatic axonal injury, *TBI* traumatic brain injury, *SD* standard deviation

* GOSE score was obtained 12 months after injury. 7 of the GOSE scores are from 6 months due to missing GOSE score at 12 months. 22 GOSE scores were imputed due to missing GOSE scores at both time points, thus n=65 instead of 60 as in Table 1 where imputed values are not included (see Materiel and Methods)

** Comparing all the outcome groups

ᵻ Severe TBI: GCS ≤ 8. For 3 patients exact GCS score is missing but we were able to classify HISS category

# Including corpus callosum, internal capsule, thalamus and basal ganglia

After adjusting for age, pupil abnormality, GCS score and number of days from injury to MRI, both total brain contusion volume and hemorrhagic brain contusion volume were significantly associated with severe disability in all multivariable models: models 1 and 3 included the Trondheim TAI-MRI grade, and models 2 and 4 included the TAI volume on FLAIR (Table 4). In the VLSM analysis, there was no significant association between anatomical lesion distribution and the GOSE score in any of the voxels.

**Table 4:** Multivariable ordinal logistic regression models predicting 12-month outcome categories* including total (models 1 and 2) and hemorrhagic (models 3 and 4) brain contusion volumes

	Model 1		Model 2		Model 3		Model 4	
	OR (95% CI)	*p*-value	OR (95% CI)	*p*-value	OR (95% CI)	*p*-value	OR (95% CI)	*p*-value
Age	1.06 (1.04–1.07)	**< 0.001**	1.05 (1.03–1.07)	**< 0.001**	1.06 (1.04–1.08)	**< 0.001**	1.06 (1.04–1.08)	**< 0.001**
Pupil abnormality	2.31 (1.17–4.57)	**0.02**	2.69 (1.39–5.24)	**0.004**	2.75 (1.41–5.37)	**0.003**	3.06 (1.59–5.90)	**0.001**
GCS score**	1.40 (1.27–1.53)	**< 0.001**	1.40 (1.27–1.54)	**< 0.001**	1.41 (1.28–1.55)	**< 0.001**	1.41 (1.28–1.55)	**< 0.001**
Days from injury to MRI***	1.01 (0.98–1.03)	0.63	1.00 (0.98–1.03)	0.87	1.01 (0.98–1.03)	0.48	1.01 (0.98–1.03)	0.64
Total lesion volume of TAI on FLAIR-MRI (cm^3^)	1.10 (1.04–1.16)	**< 0.001**			1.09 (1.04–1.15)	**0.001**		
Trondheim TAI-MRI grade			1.26 (1.05–1.52)	**0.02**			1.26 (1.04–1.52)	**0.02**
Total lesion volume of brain contusion on FLAIR-MRI (cm^3^)	1.01 (1.01–1.02)	**< 0.001**	1.01 (1.01–1.02)	**< 0.001**				
Total volume of hemorrhagic component of brain contusion in FLAIR-MRI (cm3)					1.03 (1.00–1.06)	**0.02**	1.03 (1.00–1.06)	**0.02**
Model fit (R^2^)	0.29		0.27		0.27		0.25	

Normal pupils were set as reference category for pupil abnormality. The variable for GOSE category was inverted and analyzed as a dependent variable; thus, the models are predicting worse outcome category. Values are given in odds ratios (ORs) and 95% confidence intervals (CIs). Model fit is given as McFadden’s pseudo R^2^. *p*-values under 0.05 are indicated in bold

*GOSE* Glasgow outcome scale extended, *GCS* Glasgow coma scale, *MRI* magnetic resonance imaging, *FLAIR* fluid-attenuated inversion recovery, *TAI* traumatic axonal injury, *GOSE* Glasgow coma scale extended

* All models predict GOSE category (GOSE 1–4, GOSE 5–6 and GOSE 7–8) with multiple ordinal logistic regression, with the indicated variables as covariates. GOSE score was obtained 12 months after injury. Seven of the GOSE scores are from 6 months due to missing GOSE score at 12 months. 22 GOSE scores were imputed due to missing GOSE scores at both time points

** The GCS score was inverted to obtain interpretable odds ratios

*** Days from injury to MRI were significantly associated with outcome category in an univariable ordinal logistic regression model (*p* = 0.02)

## Discussion

In this large prospective study, segmentations of brain contusions on early MRI from patients with moderate or severe TBI were analyzed visually on lesion frequency distribution maps and with conventional statistics. Brain contusions were most common in the frontal and temporal lobes. When moderate and severe TBI were compared, we found no significant differences in the distribution or total volume of brain contusions. There was a trend toward a decreasing number and volume of total brain contusions with a better outcome category, and in adjusted models, brain contusion volume predicted severe disability at 12 months. In the VLSM analyses, we did not find any significant association between the anatomical distribution of brain contusions and the 12-month outcome.

### Frequency and distribution of brain contusions in moderate and severe TBI combined

In the present study, we found that the inferior and rostral parts of the temporal and frontal lobes were predilection sites for brain contusions. This is in accordance with both previous MRI studies [1, 2, 15] and pathological studies [3]. We found similar distribution patterns for hemorrhagic and non-hemorrhagic components of the brain contusions. An earlier small MRI study (*n* = 23) on brain contusions using lesion frequency distribution maps also revealed a rostral frontal and temporal distribution pattern when analyzing patients with all severities of TBI and MRI obtained at 1 year [15]. The compressive forces on the frontal and temporal lobes against the uneven skull base of the anterior and middle cranial fossa, combined with the higher amplitudes of cortical brain shift observed in these regions [36], may explain why the frontal and temporal lobes are predilection sites for brain contusions.

### Frequency and distribution of brain contusions in moderate versus severe TBI

Surprisingly, we found no significant difference in total brain contusion volume between patients with moderate and severe TBI, and patients with moderate TBI had significantly larger hemorrhagic volumes. These findings appear counterintuitive but might be influenced by several factors. First, our classification of injury severity relies solely on the GCS score. Even though the GCS remains the most commonly used tool for TBI classification [37], it is unidimensional and does not capture the full heterogeneity of TBI [38]. For instance, patients with severe TBI had significantly higher TAI grades than those with moderate TBI. Our research group has previously shown that there is a strong association between TAI burden and the GCS score [24]. Injury severity, classified by the GCS score, may therefore be more strongly associated with the TAI grade than with the brain contusion volume.

Second, the patients with moderate TBI were older and more often suffered falls, which is known to be associated with a greater risk of brain contusions [39, 40]. Third, since the patients with moderate TBI obtained MRI significantly earlier than those with severe TBI, the smaller hemorrhagic volume in severe TBI could be due to some resorption of hemorrhage. However, we did not find any correlation between the number of days from injury to MRI and hemorrhagic brain contusion volume in our dataset.

Nonetheless, our finding that total brain contusion volume is not associated with injury severity corresponds with previous MRI studies showing no association between the GCS score and the presence [41] or total volume of brain contusions [16].

### Prognostic value of brain contusions on MRI

When comparing patients with severe disability, moderate disability and good recovery, we demonstrated decreased brain contusion frequency with improved outcome categories. However, most of the patients with severe disability had sustained severe TBI and had high TAI grades, which likely also contributed to their outcomes [24]. After adjusting for age, pupil abnormality, GCS score, number of days from injury to MRI and (1) Trondheim TAI grade and (2) TAI volume on FLAIR in the four different models, we found that both total and hemorrhagic brain contusion volumes were significantly associated with the outcome category in moderate and severe TBI combined. The model including the total volume of brain contusions and the TAI had the best overall model fit.

Despite the statistical significance, the odds ratios were modest, and other types of pathologies, such as TAI, may have greater prognostic value. Therefore, total and hemorrhagic brain contusion volumes need to be considered with other clinical and imaging variables. Our research group has previously shown that patients with severe TBI have significantly more TAI lesions, larger TAI volumes and higher grades of TAI than patients with moderate TBI [24]. This, together with the finding that brain contusions only have prognostic value in moderate TBI [13], indicates that TAI, to a greater extent than brain contusions, is associated with injury severity and outcome.

The VLSM analyses revealed no significant associations between the presence of brain contusions at any location and outcomes measured by the GOSE at 12 months, indicating that the functional outcome after moderate-severe TBI is independent of the brain contusion location at the voxel level and that the brain contusion volume is more important. We therefore suggest that brain contusion volume on MRI, either total or hemorrhagic, should be included in future multidimensional prognostic models for TBI.

### Strengths and limitations

A comprehensive set of prospective data from a large MRI cohort of moderate and severe TBI patients is a strength of this study. We also performed high-quality segmentation of the brain contusions performed by radiologists, all of whom were blinded to clinical information and patient identification, with high interrater agreement.

The limitations of the study include first that the patients underwent MRI at different stages of injury (0–42 days post-injury), which potentially gives heterogeneity in lesion characteristics. Brain contusions may change over time due to both progression and resorption of the lesions [21, 23]. These effects could, to some degree, impact both the observed distribution and the measured volumes. However, we did not find any association between the number of days from injury to MRI and total brain contusion volume in our dataset when we adjusted for age and GCS score. Ideally, MRI should be obtained at the same time point post-injury for all patients, but this is not possible in a clinical cohort of moderate-severe TBI patients due to both medical and logistical reasons. Second, different MRI scanners and different field strengths were used. Compared to the other patients, the seven patients examined with 1-T scanners may have had underestimated brain contusion volumes. However, the majority of the patients were examined with 1.5-T scanners (86%), and MRI at 1–3 T is significantly more sensitive for brain contusions than CT [41–43].

Third, a limitation of the VLSM analysis is that our data most likely do not fulfill the normality assumption of the *t*-test, particularly due to the ordinal nature of the GOSE score. While the results of the test are still valid, this reduces the sensitivity, and an alternative nonparametric test might have uncovered weak associations [44]. However, none of the VLSM tools available to us have implemented such tests, and we were therefore not able to use them in this study.

## Conclusions

In this large prospective study of patients with moderate-severe TBI and early MRI findings, we demonstrated in lesion frequency distribution maps that brain contusions were most common in the frontal and temporal lobes. There was no significant difference in the distribution or frequency of total brain contusion volume between moderate and severe TBI patients. We found a trend toward increasing total brain contusion volume with worsening outcome categories, and adjusted models showed that both total and hemorrhagic brain contusion volumes predict severe disability. With voxel-based analyses, we did not find any significant association between the location of the brain contusions and the 12-month outcome, indicating that the volume of the brain contusions is more important for functional outcomes than the specific lesion location.

## Supplementary information

ELECTRONIC SUPPLEMENTARY MATERIAL

Video 1

Video 2

Video 3

## Abbreviations

FLAIR: Fluid-attenuated inversion recovery
GOSE: Glasgow outcome scale-extended
TAI: Traumatic axonal injury
TBI: Traumatic brain injury
VLSM: Voxel-based lesion-symptom mapping
