# Supplementary material for: Moderate and severe traumatic brain injury: lesion frequency distribution maps and prognostic importance of brain contusions on early MRI
Source: Eur Radiol. 2025 Dec 19;36(5):3518–30. doi: 10.1007/s00330-025-12159-y (PMC13086803; doi:10.1007/s00330-025-12159-y)
Supplement: Supplementary file 1 — ELECTRONIC SUPPLEMENTARY MATERIAL [file 330_2025_12159_MOESM1_ESM.pdf]

Moderate and severe traumatic brain injury: Lesion frequency distribution maps and prognostic importance of brain contusions on early MRI

ELECTRONIC SUPPLEMENTARY MATERIAL

Eur Radiol (2025) Eliassen TS, Bø LE, Flusund AMH, et al.

Supplementary material and methods

Patients

The Trondheim cohort consisted of all patients admitted to St.Olav's University Hospital in Trondheim between October 2004 and October 2021 with moderate (Glasgow coma scale (GCS) 9–13) or severe (GCS ≤ 8) traumatic brain injury (TBI), according to the Head Injury Severity scale (HISS) [1]. *The Oslo cohort* consisted of all patients ≥ 16 years admitted to Oslo University Hospital between January 2009 and January 2011 with a GCS score ≤ 8 at some time points within the first 24 hours.

Exclusion criteria

The exclusion criteria were (figure 1):

1. age > 70 years because of a higher expected prevalence of age-related white matter hyperintensities [2] and non-traumatic brain pathology,
2. age < 8 years because the Glasgow Outcome Scale Extended (GOSE) adult version is less suited for the assessment of small children [3],
3. MRI obtained > 6 weeks after injury due to attenuation of some traumatic lesions over time [4], and
4. unreadable MRI due to poor quality, large artefacts, or acute large-vessel ischemia/infarction.

Classification of CT findings

Head CT examinations of all patients were reviewed by radiologists or residents in radiology in collaboration with neuroradiologists for *the Trondheim cohort* [5] and by neuroradiologists for *the Oslo cohort* [6]. A predefined scheme including registration of the first and worst Marshall [7] and Rotterdam CT scores [8] was applied.

Neuroimaging and MRI reading

In *the Trondheim cohort* MRI was obtained at St. Olav's hospital, Trondheim University Hospital or one of the local hospitals in the corresponding health regions. In *the Oslo cohort* MRI was obtained at Oslo University Hospital.

The MRIs were reviewed by six senior consultants in radiology (KGM, AMHF, EHS, ØO, SAG, AKK) and one resident in radiology (GGG, n=13) in collaboration with KGM. Everyone was blinded to the clinical information and patient identification.

Brain contusions were defined as focal lesions with edema (non-hemorrhagic brain contusions) and often hemorrhage (hemorrhagic brain contusions) located in the cerebral cortex and/or subcortical white matter, with the bulk of the lesion close to the skull or dural folds. Traumatic parenchymal hemorrhages were defined as intraparenchymal hemorrhages with a homogeneous hemorrhagic component > 10 mm on FLAIR [9] and often with minor surrounding edema. Hemorrhagic brain contusions can, in a few cases, be difficult to distinguish from traumatic parenchymal hemorrhages [10]. In the present study, two patients had traumatic parenchymal hemorrhages. For simplicity we included these two in the brain contusion group.

In addition to the lesion measures, probable affected eloquent cortical areas, defined as neurological functioning areas in which the injury may have resulted in disability because of the anatomical location of the lesions on MRI, were registered. The eloquent areas were divided into the motor cortex (gyrus pre-centralis), sensory cortex (gyrus post-centralis), visual cortex (occipital cortex), speech area (left posterior part of gyrus frontalis inferior, left posterior part of the gyrus temporalis superior or the arcuate fasciculus) and primary auditive cortex (Heschl's gyrus). The probable affected primary auditive cortex was only registered if the lesion was bilateral since unilateral lesions usually have no clinical relevance [11].

Interrater agreement for brain contusions

A total of 30 MRI scans scored by KGM were randomly selected from *the Trondheim cohort* and independently rescored by AMHF, EHS and ØO. Interrater agreement for the total volume of brain contusions on fluid attenuated inversion recovery (FLAIR) was assessed and presented in a previous paper [9]. The intraclass correlation coefficient (ICC) was 0.85, indicating good agreement [12].

Imputation of missing GOSE scores

15 of the patients with missing GOSE scores were included in a related study, and more details on the imputation of GOSE scores and sensitivity analyses for these patients can be found in a previous paper [13]. Imputation of GOSE scores for the 7 remaining patients was done with the same approach, with the inclusion of the following covariables: age, pupil size, GCS score, Marshall scores, presence and total volume of brain contusions, total volume of parenchymal hemorrhage, total volume of TAI on FLAIR, DWI and SWI and standard TAI grade.

Statistics

A one-way ANOVA model was used to explore the associations between the log-transformed brain contusion volume and the MRI field strength. The Tuckey Honestly Significant Difference (HSD) test was used for post hoc analyses. To explore the adjusted relationship between total brain contusion volume and MRI field strength we performed a linear regression model with post-hoc analysis using log-transformed total brain contusion volume as the dependent variable, and MRI field strength, GCS score and evacuation of mass lesions as covariates. A general linear model was used for post hos analyses.

The Spearman rank correlation test was performed to explore the associations between the number of days between injury and MRI findings and both total and hemorrhagic brain contusion volume. To adjust for age and GCS score, we further performed a linear regression model with log-transformed total brain contusion volume as the dependent variable and the number of days from injury to MRI, age and GCS score as covariates. Ideally, we would have performed a linear regression model with the log-transformed hemorrhagic brain contusion volume as

well, but since several patients had a hemorrhagic brain contusion volume of zero, log transformation was not feasible. Since the Spearman correlation was non-significant, we did not find it necessary to perform adjusted analyses for this correlation.

Data collection

Data collection was performed using a web-based data collection system developed and administered by the faculty of Medicine and Health Sciences, Norwegian University of Science and Technology, Trondheim, Norway.

Supplementary tables

Supplementary table 1: Lesion distribution of brain contusions on early MRI in the brain lobes based on individual segmentations in moderate and severe TBI patients combined.

Number of patients with brain contusion(s) on FLAIR-MRI in:	Moderate and severe TBI*, n=301	*p*-value**
Right frontal lobe	43 (14%)	**<0.001**
Left frontal lobe	63 (21%)	
Both frontal lobes	120 (40%)	
Right temporal lobe	66 (22%)	0.76
Left temporal lobe	85 (28%)	
Both temporal lobes	95 (32%)	
Right parietal lobe	31 (10%)	**0.004**
Left parietal lobe	24 (8%)	
Both parietal lobes	11 (4%)	
Right occipital lobe	13 (4%)	**0.01**
Left occipital lobe	7 (2%)	
Both occipital lobes	3 (1%)	

Values are presented as number of patients (%) or median (IQR) unless otherwise is indicated. *p*-values under 0.05 are indicated in bold.
Abbreviations: FLAIR = fluid attenuated inversion recovery, MRI = magnetic resonance imaging, TBI = traumatic brain injury, GCS = Glasgow coma scale, HISS = head injury severity scale.
*Severe TBI: GCS ≤ 8 and moderate TBI: GCS 9–13. For 5 patients exact GCS score is missing but we were able to classify HISS category.
**Comparing right lobe to left lobe.

Supplementary table 2: One-way ANOVA* with log-transformed total brain contusion volume as dependent variable and MRI field strength as the factor.

MRI field strength		Mean difference (95% CI)	*p*-value
1.0 T (n=7)	1.5 T	-2.36 (-3.98 – -0.74)	**0.002**
	3.0 T	-2.45 (-4.20 – -0.71)	**0.003**
1.5 T (n=258)	1.0 T	2.36 (0.74 – 3.98)	**0.002**
	3.0 T	-0.10 (-0.85 – 0.65)	0.95
3 T (n=36)	1.0 T	2.45 (0.71 – 4.20)	**0.003**
	1.5 T	0.10 (-0.65 – 0.85)	0.98

*The one-way ANOVA determined a statistical difference in log-transformed total brain contusion volume between the three different MRI field strengths (F(2,298)=5.996, ***p*=0.003**). The table shows the results from the Tuckey Honestly Significant Difference (HSD) Post Hoc Test.
P-values under 0.05 are indicated in bold.
Abbreviations: MRI = magnetic resonance imaging, CI = confidence interval, T = tesla.

Supplementary table 3: Linear regression and post hoc test with log-transformed total brain contusion volume as the dependent variable and MRI field strength, GCS score and evacuation of mass lesions as covariates.

Linear regression model

Variables	Unstandardized coefficient (95% CI)	*p*-value
MRI field strength	0.64 (0.06–1.22)	**0.03**
GCS score	0.02 (-0.04–0.08)	0.42
Evacuation of mass lesion	0.88 (0.39–1.38)	**<0.001**

Post hoc test

MRI field strength		Mean difference (95% CI)	*p*-value
1.0 T (n=7)	1.5 T	-2.47 (-4.37 – -0.57)	**0.01**
	3.0 T	-2.43 (-4.53 – -0.33)	**0.02**
1.5 T (n=258)	1.0 T	2.47 (0.57 – 4.37)	**0.01**
	3.0 T	0.04 (-0.95 – 1.02)	0.94
3 T (n=36)	1.0 T	2.43 (0.33 – 4.53)	**0.02**
	1.5 T	-0.04 (-1.02 – 0.95)	0.94

p-values under 0.05 are indicated in bold.
Abbreviations: MRI = magnetic resonance imaging, GCS = Glasgow coma scale, CI = confidence interval, T = tesla.

Supplementary table 4: Spearman rank test for correlation between brain contusion volume and the number of days from injury to MRI.

Correlation between	Spearmans correlation coefficient (95% CI)	*p*-value
Total brain contusion volume and number of days from injury to MRI	0.14 (0.03–0.25)	**0.01**
Hemorrhagic brain contusion volume and number of days from injury to MRI	0.09 (-0.02–0.21)	0.10

p-values under 0.05 are indicated in bold.
Abbreviations: MRI = magnetic resonance imaging, CI = confidence interval.

Supplementary table 5: Linear regression with log-transformed total brain contusion volume as the dependent variable and age, GCS score and number of days from injury to MRI as covariates.

Variables	Unstandardized coefficient (95% CI)	*p*-value
Age	0.03 (0.02–0.045)	**<0.001**
GCS score	0.00 (-0.06–0.06	0.92
Days from injury to MRI	0.02 (-0.00–0.04)	0.07

p-values under 0.05 are indicated in bold.
Abbreviations: GCS = Glasgow coma scale, MRI = magnetic resonance imaging, CI = confidence interval.

Video 1: *Lesion frequency distribution maps* of total brain contusion versus hemorrhagic component on FLAIR

Video scrolling through the *lesion frequency distribution maps* showing the relative frequency of cases with total brain contusion (left) and a hemorrhagic component (right) in the transverse plane. The color scale ranges from blue to red where dark blue indicates at least one patient per voxel (~1%), and the darkest shade of red indicates a high percentage (up to 20%) of patients per voxel.
FLAIR = fluid attenuated inversion recovery, n = number.

Video 2: *Lesion frequency distribution maps* of brain contusions on FLAIR in severe versus moderate TBI

Video scrolling through the *lesion frequency distribution maps* showing the relative frequency of brain contusions in patients with severe TBI (left) and moderate TBI (right) in the transverse plane. The color scale ranges from blue to red, where dark blue indicates at least one patient per voxel (~1%), and the darkest shade of red indicates a high percentage (~20%) of patients per voxel.
FLAIR = fluid attenuated inversion recovery, TBI = traumatic brain injury, n = number.

Video 3: *Lesion frequency distribution maps* of brain contusions on FLAIR in different 12-month outcome categories

Video scrolling through the *lesion frequency distribution maps* showing the relative frequency of brain contusions in patients with severe disability (GOSE score of 1–4, top left), moderate disability (GOSE score of 5–6, top right) and good recovery (GOSE score of 7–8, bottom left) in the transverse plane. The color scale ranges from blue to red where dark blue indicates at least one patient per voxel (~1%), and the darkest shade of red indicates a high percentage (~20%) of patients per voxel.
FLAIR = fluid attenuated inversion recovery, GOSE = Glasgow outcome scale extended, n = number.

Supplementary references

1 Stein SC, Spettell C (1995) The Head Injury Severity Scale (HISS): a practical classification of closed-head injury. Brain Inj 9:437–444
2 de Leeuw FE, de Groot JC, Achten E et al (2001) Prevalence of cerebral white matter lesions in elderly people: a population based magnetic resonance imaging study. The Rotterdam Scan Study. J Neurol Neurosurg Psychiatry 70:9–14
3 Beers SR, Wisniewski SR, Garcia-Filion P et al (2012) Validity of a pediatric version of the Glasgow Outcome Scale-Extended. J Neurotrauma 29:1126–1139
4 Moen KG, Skandsen T, Folvik M et al (2012) A longitudinal MRI study of traumatic axonal injury in patients with moderate and severe traumatic brain injury. J Neurol Neurosurg Psychiatry 83:1193–1200
5 Moe HK, Limandvik Myhr J, Moen KG, Håberg AK, Skandsen T, Vik A (2019) Association of cause of injury and traumatic axonal injury: a clinical MRI study of moderate and severe traumatic brain injury. J Neurosurg. 10.3171/2019.6.Jns191040:1–9
6 Roe C, Skandsen T, Anke A et al (2013) Severe traumatic brain injury in Norway: impact of age on outcome. J Rehabil Med 45:734–740
7 Marshall LF, Marshall SB, Klauber MR et al (1991) A new classification of head injury based on computerized tomography. Journal of neurosurgery 75:S14–S20
8 Maas AI, Hukkelhoven CW, Marshall LF, Steyerberg EW (2005) Prediction of outcome in traumatic brain injury with computed tomographic characteristics: a comparison between the computed tomographic classification and combinations of computed tomographic predictors. Neurosurgery 57:1173–1182; discussion 1173–1182
9 Moe HK, Follestad T, Andelic N et al (2020) Traumatic axonal injury on clinical MRI: association with the Glasgow Coma Scale score at scene of injury or at admission and prolonged posttraumatic amnesia. J Neurosurg. 10.3171/2020.6.Jns20112:1–12
10 Kim JJ, Gean AD (2011) Imaging for the Diagnosis and Management of Traumatic Brain Injury. Neurotherapeutics 8:39–53
11 Mendoza JE (2017) Heschl's Gyrus. Springer International Publishing, pp 1–1
12 Hallgren KA (2012) Computing Inter-Rater Reliability for Observational Data: An Overview and Tutorial. Tutor Quant Methods Psychol 8:23–34
13 Flusund A-MH, Bø LE, Reinertsen I et al (2024) Lesion Frequency Distribution Maps of Traumatic Axonal Injury on Early Magnetic Resonance Imaging After Moderate and Severe Traumatic Brain Injury and Associations to 12 Months Outcome. Journal of Neurotrauma. 10.1089/neu.2023.0534
